# Exploration of the Potential Link, Hub Genes, and Potential Drugs for Coronavirus Disease 2019 and Lung Cancer Based on Bioinformatics Analysis

**DOI:** 10.1155/2022/8124673

**Published:** 2022-09-26

**Authors:** Ye Wang, Qing Li, Jianfang Zhang, Hui Xie

**Affiliations:** ^1^Department of Thoracic Surgery, Affiliated Hospital (Clinical College) of Xiangnan University, Chenzhou 423000, Hunan Province, China; ^2^School of Medical Imaging, Laboratory Science and Rehabilitation, Xiangnan University, Chenzhou 423000, Hunan Province, China; ^3^Key Laboratory of Medical Imaging and Artificial Intelligence of Hunan Province, 423000 Chenzhou, Hunan Province, China; ^4^Department of Physical Examination, Beihu Centers for Disease Control and Prevention, Chenzhou 423000, Hunan Province, China; ^5^Department of Radiation Oncology, Affiliated Hospital (Clinical College) of Xiangnan University, Chenzhou 423000, Hunan Province, China

## Abstract

The ongoing pandemic of coronavirus disease 2019 (COVID-19) has a huge influence on global public health and the economy. Lung cancer is one of the high-risk factors of COVID-19, but the molecular mechanism of lung cancer and COVID-19 is still unclear, and further research is needed. Therefore, we used the transcriptome information of the public database and adopted bioinformatics methods to identify the common pathways and molecular biomarkers of lung cancer and COVID-19 to further understand the connection between them. The two RNA-seq data sets in this study—GSE147507 (COVID-19) and GSE33532 (lung cancer)—were both derived from the Gene Expression Omnibus (GEO) database and identified differentially expressed genes (DEGs) for lung cancer and COVID-19 patients. We conducted Gene Ontology (GO) functions and Kyoto Encyclopedia of Genes and Genomes (KEGG) pathways enrichment analysis and found some common features between lung cancer and COVID-19. We also performed TFs-gene, miRNAs-gene, and gene-drug analyses. In total, 32 DEGs were found. A protein-protein interaction (PPI) network was constructed by DEGs, and 10 hub genes were screened. Finally, the identified drugs may be helpful for COVID-19 treatment.

## 1. Introduction

Lung cancer (LC) is a malignant tumor originating from the trachea, bronchial mucosa, and glands. Globally, LC morbidity and mortality are extremely high and on the rise [1]. The morbidity and mortality of LC ranks first among all malignant tumors for the male population, while the LC morbidity ranks third and the LC mortality ranks second for the female population [1]. LC treatment includes long-term chemotherapy cycles and drug treatments. The FDA-approvedanti-LC drugs include crizotinib, atezolizumab, pembrolizumab, pemetrexed, bevacizumab, and so on [2]. Coronavirus disease 2019 (COVID-19) that is also a lung disease with strong and widespread transmissibility, and general susceptibility, has had a significant impact on the health and economies of people around the world. According to the report of World Health Organization (WHO), as of March 4, 2022, there were a total of 441,700,000 confirmed cases of COVID-19 worldwide, with 6,000,000 deaths [3]. The currently recommended anti-COVID-19 drugs include chloroquine, hydroxychloroquine (HCQ), lopinavir/ritonavir, ribavirin, oseltamivir, remdesivir, favipiravir, and umifenovir [4]. Since COVID-19 is severe and highly contagious, the population is generally susceptible. Among the confirmed cases, LC patients have weaker physical immunity and are more susceptible to infection [5]. Studies have found that, compared with noncancer patients, cancer patients are at higher risk of COVID-19 and have a poorer prognosis, and lung cancer patients are the most common [6].

There is no direct evidence that COVID-19 can transform into LC. However, long-term repeated chronic pneumonia can affect the function of the bronchial mucosal epithelium and the body's immunity [7]. Therefore, it has an indirect promotion effect on the occurrence of LC. In contrast, studies have confirmed that LC can cause pneumonia [8]. Some tumors can cause bronchial obstruction, leading to poor drainage of the distal lungs and forming obstructive pneumonia [9]. COVID-19 is currently considered to be an immune self-limiting disease to a certain extent [10] and therefore closely linked to immune function. A study [11] also found that CD4+ and CD8+ T lymphocyte subsets were significantly reduced in COVID-19 patients. When malignant tumors appear in the body, the ratio of CD4+/CD8+ also decreases significantly, and lung cancer patients are in a state of immunosuppression [12], which makes lung cancer patients susceptible to COVID-19. A retrospective study by Yu et al. [13] found that the incidence of COVID-19 infection in tumor patients was 0.79%, which was 2.31 times that of the general population. It is very interesting to report that a patient with Hodgkin's lymphoma developed extensive tumor regression after infection with COVID-19 [14]. A similar case also appeared in the field of LC. Therefore, we hypothesize that there may be some links between COVID-19 and LC.

**Figure 1:**
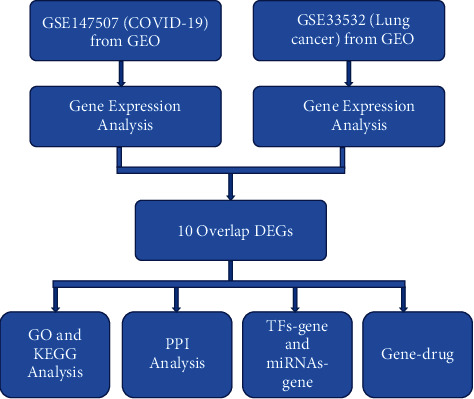
The flow chart of this study.

This study aimed to explore the link between the COVID-19 and LC using the LC and COVID-19 data sets from the Gene Expression Omnibus (GEO, https://www.ncbi.nlm.nih.gov/geo) based on bioinformatics analysis. The biological functions of COVID-19-LC related hub genes were analyzed. Finally, recommendations were made for potential chemical drugs. The flow chart of this study is shown in Figure 1.

## 2. Materials and Methods

### 2.1. Data Collection

The microarray data of GSE147507 [15] and GSE33532 [16] were downloaded from the GEO. In the GSE147507 data set, there are 55 samples that are not infected with COVID-19 and 23 samples that are infected with COVID-19. In the GSE33532 data set, there are 497 LC samples and 54 normal lung samples.

### 2.2. Identification of the Overlapping Differentially Expressed Genes between COVID-19 and LC

The DESeq2 package of *R* was used to perform gene expression analysis, thereby identifying the differentially expressed genes (DEGs) in the GSE147507. In addition, the limma package of *R* was used to identify DEGs in the GSE33532. The filtering criteria for DEGs were |logFold Change (FC)| ≥2 and the false discovery rate (FDR) <0.05. The overlapping DEGs between GSE147507 and GSE33532 were generated using the Venny package of *R*.

### 2.3. Gene Ontology Functions and Kyoto Encyclopedia of Genes and Genomes Pathways Enrichment Analyses

Gene Ontology (GO) functions and Kyoto Encyclopedia of Genes and Genomes (KEGG) pathways enrichment analyses were performed using the “org.Hs.eg.db, cluster Profiler, and enrichplot” packages of *R*. GO terms were considered significant if the adjusted Benjamini–Hochberg corrected *P*-value was less than 0.05. KEGG pathways with corrected *P*-value less than 0.05 were considered significantly enriched by DEGs.

### 2.4. Construction of Protein-Protein Interaction Network and Identification of the Hub Genes

The Search Tool for the Retrieval of Interacting Genes/Proteins (STRING; version 11.0, https://string-db.org/) was used to obtain the protein-protein interaction (PPI) network based on the overlapping DEGs. In this present study, the minimum required interaction score was 0.15. The top 10 hub genes were picked by the maximal clique centrality (MCC) using Cytoscape plugin cytoHubba.

### 2.5. Identification of Transcription Factors and miRNAs Binding to Overlapping DEGs

The significant transcription factors (TFs) were identified using a freely accessible database of TFs repository-JASPAR (https://www.networkanalyst.ca). These TFs tend to bind to the overlapping DEGs. Through the interaction of miRNAs-gene via network analysts, we had picked out miRNAs from both TarBase and miRTarBase that interact with overlapping DEGs focused on topological analysis.

### 2.6. Identification of Potential Drugs for Both COVID-19 and LC

We used protein-drug interaction data from the Drug SIGnatures database (DSigDB, https://tanlab.ucdenver.edu/dsigdb) via Enrichr to identify potential drugs to be proposed in COVID-19 and LC based on the overlapping DEGs. Enrichr is a Web-based interactive enrichment analysis tool. The DSigDB, a drug-gene interaction database, provides information on the association of genes with their known or potential drugs. DSigDB has a total of more than 22,527 drug-gene interactions, all of which were collected and sorted through public documents and can be downloaded for free for scientific research. Expression files of overlapping genes were input into the DSigDB. The DSigDB was automatically searched to find out the significant chemical drugs for these overlapping genes.

### 2.7. Gene-Disease Association Analysis

DisGeNET (https://www.disgenet.org/) is a very comprehensive database about gene-disease associations. It synchronizes relationships from several origins with various biomedical aspects of diseases. It focuses on new insights into human genetic diseases. In this study, we also investigated the gene-disease relationship through network analysts to discover associated diseases and chronic complications associated with overlap DEGs.

## 3. Results

### 3.1. Identification of the Overlapping DEGs between COVID-19 and LC

**Figure 2:**
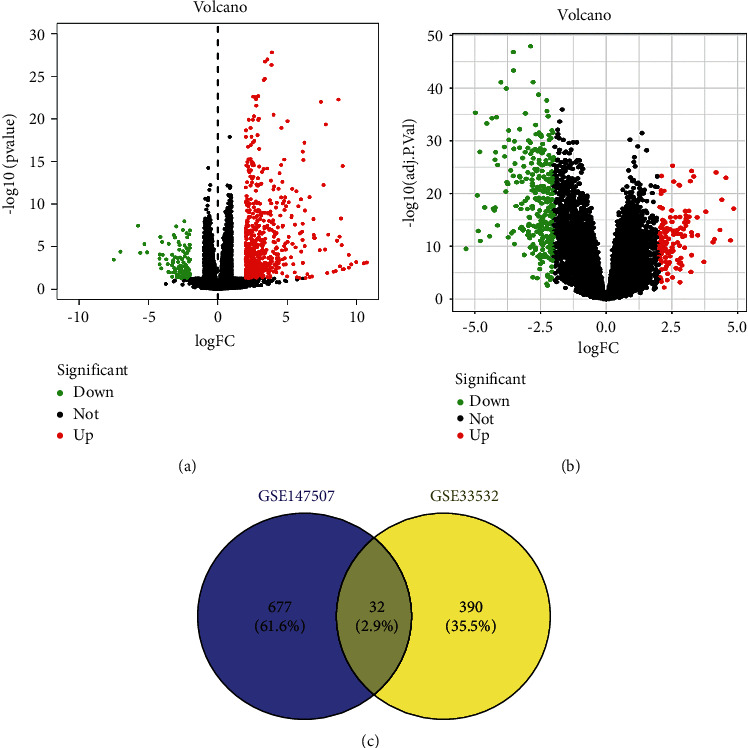
Identification differentially expressed genes (DEGs): (a) the volcano plot of DEGs in GSE147507 (COVID-19), (b) the volcano plot of DEGs in GSE33532 (lung cancer), and (c) the Venny plot showing the 32 overlap DEGs between COVID-19 and lung cancer.

In order to explore the relationship and implication of COVID-19 and LC, we analyzed the RNA-seq and microarray data sets from GEO. The DESeq2 package of *R* was used to analyze the COVID-19 data set, and the screening criteria were |logFC| ≥2 and FDR <0.05, the results of which showed 709 DEGs, including 109 upregulated genes and 600 downregulated genes (Figure 2(a)). The limma package of *R* was used to analyze the LC data set, and the screening criteria were also |logFC| ≥2 and FDR <0.05, the results of which showed 422 DEGs, including 139 upregulated genes and 283 downregulated genes (Figure 2(b)). The Venny package of *R* was then used to identify the overlapping DEGs between COVID-19 and LC, the results of which showed 32 overlapping DEGs that were identified as potential target genes (Figure 2(c)).

### 3.2. GO Functions and KEGG Pathways Enrichment Analyses

**Figure 3:**
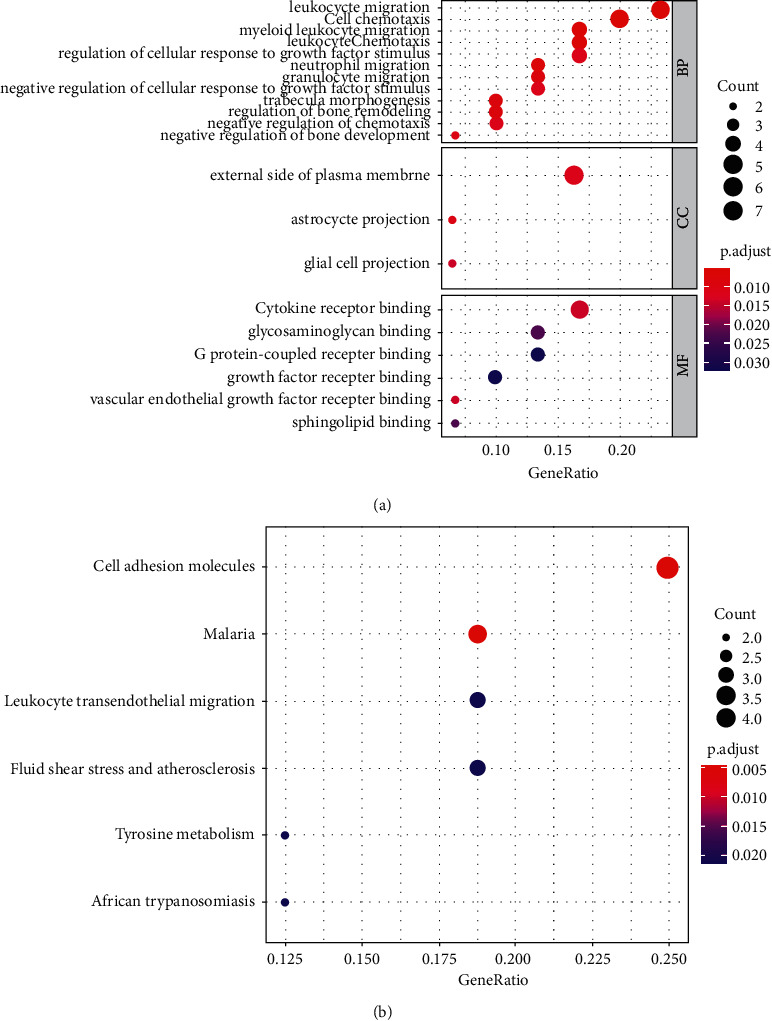
(a) GO and (b) KEGG pathways analyses of overlap DEGs.

The “org.Hs.eg.db, cluster Profiler, and enrichplot” packages of *R* were used to perform GO functions and KEGG pathways enrichment analyses for the overlapping DEGs. The GO terms included three different domains, including biological process (BP), molecular function (MF), and cellular component (CC). GO function analysis showed that the overlapping 32 genes mainly enriched in the BPs, such as “cell chemotaxis,” “leukocyte migration,” “myeloid leukocyte migration,” “leukocyte chemotaxis,” and “regulation of cellular response to growth factor stimulus,” in the CCs, such as “external side of plasma membrane,” “glial cell projection,” and “astrocyte projection,” and in the MFs, such as “cytokine receptor binding,” “vascular endothelial growth factor receptor binding,” and “sphingolipid binding” (Figure 3(a)). KEGG pathway analysis showed that the overlapping 32 genes mainly enriched in the signaling pathways, such as “cell adhesion molecules,” “leukocyte transendothelial migration,” “tyrosine metabolism,” and “fluid shear stress and atherosclerosis” (Figure 3(b)).

### 3.3. Construction of PPI Network and Identification of Hub Genes

**Figure 4:**
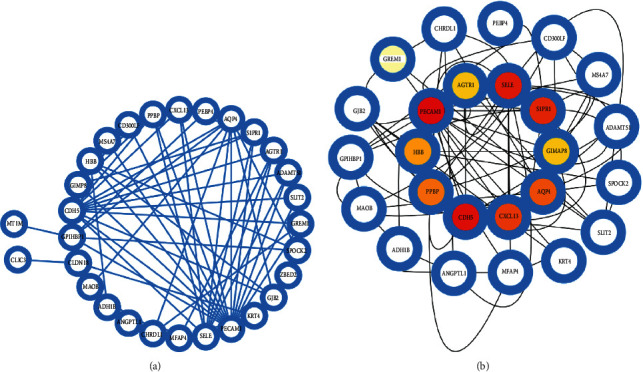
(a) PPI network of 32 overlap genes and (b) the top 10 hub genes picked out using Cytoscape plugin cytoHubba (represented by inner color circles).

The PPI network of the 32 overlapping DEGs was constructed by Cytoscape of STRING (Figure 4(a)), and this network included 32 nodes and 84 edges with an average node degree of 5.25 and average local clustering coefficient of 0.411. The top 10 hub genes were identified by cytoHubba plugin, and these hub genes were PECAM1, CDH5, SELE, S1PR1, CXC13, AQP4, PPBP, HBB, AGTR1, and GIMAP8 (Figure 4(b)). These hub genes had the highest near-connectivity and proximity in the PPI network and might become potential biomarkers, providing new therapeutic strategies for COVID-19 and LC.

### 3.4. Identification of Transcription Factors and miRNAs Binding to Overlapping DEGs

**Figure 5:**
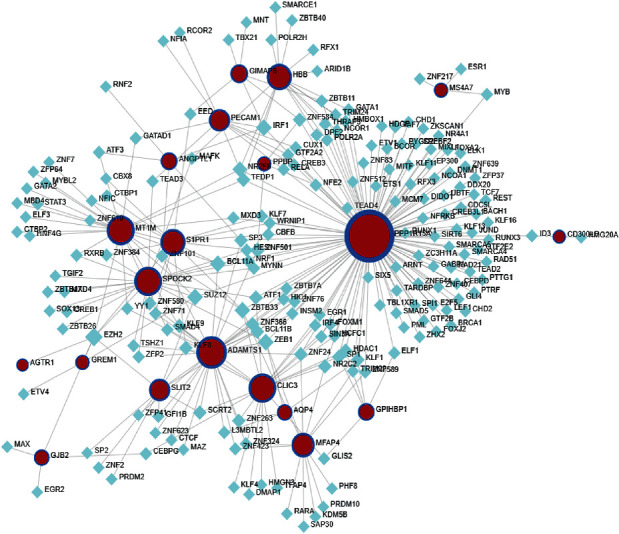
TF-genes network. TFs and genes are represented by circle and square nodes, respectively.

**Figure 6:**
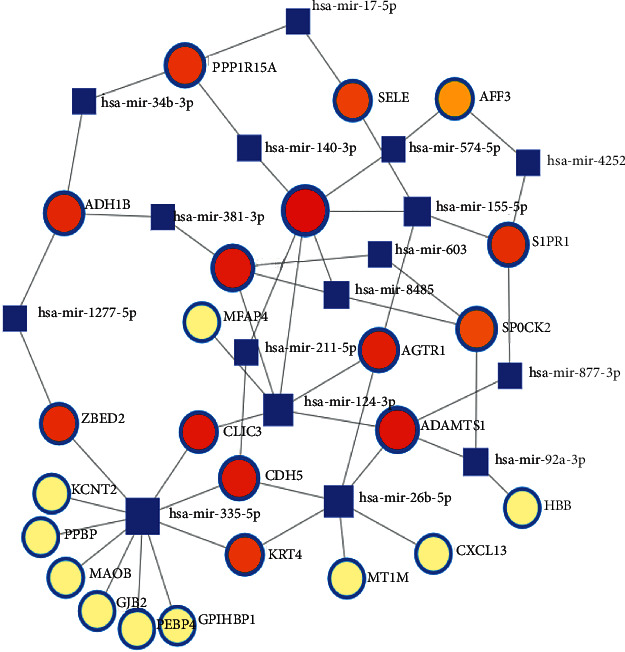
miRNAs-gene network. Genes and miRNAs are represented by circle and square nodes, respectively.

In order to identify the substantial changes that occur at the transcription level and gain insights into the regulatory molecules of central proteins or overlapping DEGs, we adopted a network-based method to decode regulatory TFs and miRNAs. The interaction between regulatory TFs and overlapping DEGs is shown in Figure 5. Similarly, Figure 6 shows the interaction of miRNA regulators with overlapping DEGs. From the analysis of the TFs-gene and miRNAs-gene interaction network, it has been determined that 27 TFs and 41 post-transcriptional (miRNAs) regulatory characteristics were regulated by more than one overlapping DEGs. This shows that there was a strong interference between them.

### 3.5. Identification of Potential Drugs

**Figure 7:**
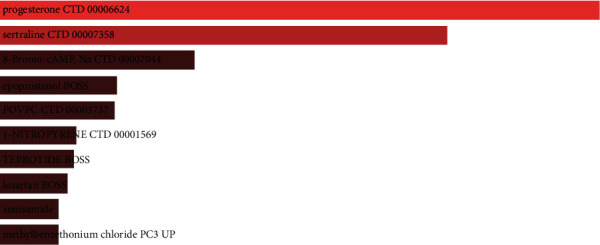
Bar diagram shows the results of drug target enrichment analysis using overlap genes as drug targets.

The study of protein-drugs interactions is critical for understanding their functions. In terms of overlapping DEGs as potential drug targets in LC and COVID-19, we used Enrichr to determine 10 possible drug molecules based on the transcriptome signature in the DSigDB. The drugs with the 10 smallest *P*-values were extracted. These 10 drugs are associated with the overlapping DEGs, and these drugs are potential drugs that may be effective against COVID-19 and LC (Figure 7).

### 3.6. Gene-Disease Association Analysis

**Figure 8:**
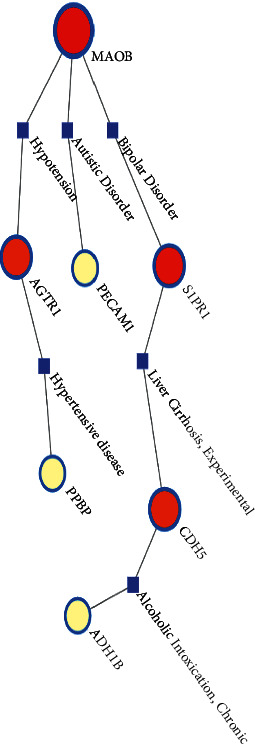
Gene-disease interaction network. Genes and diseases are represented by red and yellow nodes, respectively.

If several diseases have one or more of the same genes, then these diseases have a certain relationship that can be related to each other. This association will affect the treatment strategy of the disease. During the analysis of gene-disease (GD) associations, we found that alcoholic intoxication, liver cirrhosis, hypertensive, hypotension, bipolar disorder, and autistic disorder are the most coordinated with the pivot genes we reported, even in LC and COVID-19 are the most coordinated (Figure 8).

## 4. Discussion

Both LC and COVID-19 are lung diseases and can cause severe damage to lung function. When COVID-19 spread among the patients with LC, they are more susceptible to the COVID-19 virus than the general population [17]. Cancer patients, especially lung cancer patients, can get very bad condition once they contract COVID-19 [18]. Expression profiles of high-throughput sequencing data sets have been widely used in biomedical research and play important roles in identifying candidate biomarkers of disease. In recent years, RNA-seq technology has helped to study the status and expression differences of genes. In our study, we investigated gene expression patterns using high-throughput data sets of these two diseases based on bioinformatics analysis. Thirty-two overlapping DEGs were screened by analyzing transcriptome data sets of COVID-19 and LC. We performed GO and KEGG enrichment analysis on these overlapping DEGs to understand the biological characteristics of these overlapping DEGs in the pathogenesis of COVID-19 and LC, with a *P*-value less than 0.05. Finally, 10 hub genes that may be used as potential biomarkers of the COVID-19 were identified.

GO enrichment analysis showed that leukocyte migration, cell chemotaxis, myeloid leukocyte migration, leukocyte chemotaxis, and regulation of cellular response to growth factor stimulus were the top significantly enriched terms of BPs. Leukocyte migration is a natural process. During this process, leukocytes pass through the vascular barrier from the blood to enter the tissue in response to the invasion of pathogens [19]. This is closely related to the tumor and inflammatory microenvironment. The microenvironment affects cell phenotype [20]. It has been reported that the dysregulation of extracellular matrix dynamics may trigger the occurrence and development of tumor [21]. Due to the presence of a large number of microorganisms in pneumonia, this will also lead to an increase of leukocyte migration. The COVID-19 virus replicates massively in the body and mobilizes white blood cells to destroy the virus. In this way, leukocyte migration is very frequent. Astrocyte projection, external side of plasma membrane, and glial cell projection were the top significantly enriched terms of CCs. This suggested that DEGs might induce relevant defense responses against viruses and involve part of the nervous system. In addition, this also implied that COVID-19 and LC first have a certain destructive effect on the extracellular matrix of the lung epithelium. It was found that the *S* protein of COVID-19 can bind to the angiotensin-converting enzyme 2 (ACE2) receptor on the surface of the cell membrane to initiate the first step of infecting cells [22]. Cytokine receptor binding and vascular endothelial growth factor receptor (VEGFR) binding were the top significantly enriched terms of MFs. Soluble cytokines have been reported to prevent viral replication by inducing the expression of many interferon-stimulated genes, thereby activating antiviral and antiproliferative states [23]. It has been reported that the binding of VEGFR can promote the growth of blood vessels in lung cancer [24], thereby affecting tumor proliferation. In addition, VEGFR was recently found to be highly expressed during inflammation [25]. KEGG pathways enrichment analysis showed the most enriched pathway was cell adhesion molecules (CAMs, hsa04514). CAMs stabilize cell-cell interactions and mediate leukocyte adhesion and transendothelial migration [26]. CAMs and their receptors were found to be important in mediating inflammatory and immune responses [27]. A latest research found that the lung damage by COVID-19 was caused by inflammation [28].

The PPI network was established based on the overlapping DEGs, and the key nodes of the network play a suggestive role in the prediction of protein biological properties and drug targets. Based on the metric of this PPI network, we identified the hub proteins of COVID-19 and LC. These hub proteins were key drug targets or biomarkers for COVID-19 and also were risk factors for LC and COVID-19. These top 10 hub genes were *PECAM1, CDH5, SELE, S1PR1, CXCL13, AQP4, PPBP, HBB, AGTR1,* and *GIMAP8. PECAM1* plays an important role in the growth and stability of human organs such as the lungs and heart [29, 30]. *PECAM1* is a member of the immunoglobulin gene superfamily of adhesion molecules expressed in platelets, granulocytes, T lymphocytes, and endothelial cells [31]. Not only *PECAM1* is involved in the signal transduction of adhesion molecules and the exudation of leukocytes in inflammation, but also it acts as a connecting molecule between endothelial cells, is involved in the maintenance of endothelial cell integrity, and has a certain regulatory effect on the endothelial cell barrier function [32]. The lungs of patients with COVID-19 show unique vascular features, such as severe endothelial damage associated with cell membrane destruction [33]. The increased permeability of pulmonary microvascular endothelial cells is essentially an inflammatory response. The inflammatory response of COVID-19 pneumonia is more severe. *PECAM1* is mainly distributed in endothelial cells, located at the junctions between endothelial cells, and is involved in the regulation of leukocyte exudation and vascular permeability [34]. Endothelial cells are abundant in the lung tissue, so *PECAM1* may change in the lung tissue of COVID-19 patients and involve in COVID-19 pathological change. It has been reported that the expression of *PECAM1* in tumor tissue is highly correlated with the staging of non-small-cell LC [29]. CDH5, another hub gene, is an important hub gene in high-risk individuals for COVID-19 and LC. In addition, the vascular endothelial pathway [35] may be one of the important pathogenic mechanisms of susceptibility to COVID-19 in LC patients. The remaining hub genes further suggested that the pathogenesis of LC and COVID-19 was closely related to vascular-related signaling pathways [36–41]. *S1PR1* inhibits angiogenesis and regulates its permeability [36]. *AGTR1* [37], *AQP4* [38, 39], and *PPBP* [40] all have vascular-related functions. *E*-selectin (SELE), a member of the selectin family, is specifically expressed on the surface of stimulated endothelial cells [41]. A large amount of experimental evidence indicates that soluble SELE mainly directly mediates the adhesion of tumor cells to the vascular endothelium in tumor metastasis [42]. In the event of an inflammatory stimulus or tissue injury, the earliest response is the rolling of leukocytes along the vessel wall at the injury site. This rolling behavior is mediated by the selectin family and is a prerequisite for leukocytes to adhere to endothelial cells and to traverse the vessel wall. SELE is also involved in mediating scrolling [41]. The chemokine CXCL13 and its receptor have important roles in angiogenesis and tumor cell migration, invasion, and metastasis. When inflammation occurs, CXCL13 can also recruit *B* cells to local inflammation sites. Once infected with COVID-19, the human body undergoes a series of immune responses. When cells fail to terminate the inflammatory response, cytokine production makes macrophages hyperactive [43]. Activated macrophages destroy stem cells in the bone marrow, thereby causing anemia. This process may be related to HBB, and the abnormal expression of HBB is one of the reasons for this phenomenon [44]. GIMAP8, a member of the immune-related protein that belongs to family of GTPase, regulates lymphocyte survival and homeostasis [45]. GIMAP proteins are thought to be involved in the control of cell survival and response to infection, and GIMAP8 has been shown to have antiapoptotic functions [45]. In a study of non-small-cell lung cancer (NSCLC), it was found that GIMAP8 was abnormally expressed in tumor tissue in lower levels than in the adjacent nontumor tissue [46]. Therefore, some of these identified hub genes might be considered as potential biomarkers for COVID-19 and LC. It might also be considered as new drug targets for these two diseases.

In this study, the regulatory patterns of TFs-regulatedDEGs-miRNAs were investigated to determine whether these miRNAs are relevant to LC and COVID-19. In this way, we identified that TFs such as PPP1R15, ADAMTS, MT1M, CLIC3, SPOCK2, HBB, S1PR1, MFAP4, PECAM1, and SLIT2 are associated with LC and COVID-19. Some miRNAs (hsa-mir-335-5p, hsa-mir-26b-5p, hsa-mir-17-5p, hsa-mir-124-3p, hsa-mir-92a-3p, hsa-mir-155-5p) [47–49] are closely associated with inflammation. In addition, some miRNAs are closely related to LC, such as hsa-mir-26b-5p, hsa-mir-211-5p, and hsa-mir-155-5p [47]. Some of the miRNAs identified are strongly linked to both inflammation and LC, such as hsa-mir-26b-5p and hsa-mirr-211-5p [47, 49]. In the gene regulatory network, genes-TFs and genes-miRNAs regulate each other and regulate the expression of target genes. In this study, hsa-mir-17-5p and hsa-mir-155-5p target SLIT2, SELE, and ADAMTS1. Most of the miRNAs are associated with tumor tissue and cause LC.

The prediction results of genetic diseases (GDs) show that some diseases involved in COVID-19 include alcoholic intoxication, liver cirrhosis, hypertension, hypotension, bipolar disorder, and autistic disorder. This study found that some genes are closely related to psychiatric diseases, such as bipolar disorder and autistic disorder. Bipolar and autistic disorders are the most risk factors for dying from COVID-19. Seon et al. confirms that there is an association between SARS-CoV-2 and COVID-19 infection and the risk of death in patients with mental illness [50]. A study shows high morbidity and mortality associated with SARS-CoV-2 and COVID-19 in people with severe mental illness [51]. Shen et al. found that pneumonia was more likely to occur in older men with more severe organ damage due to hypertension, and pneumonia and LC were highly associated with mortality in urban hypertensive men [52]. The SARS-CoV-2 virus can damage the liver, and the mortality of COVID-19 patients with chronic liver disease is often high [53]. Additionally, one study reported that 2–11% of COVID-19 patients had primary chronic liver disease [54]. Liver involvement in COVID-19 patients is directly related to the cytopathic effects of the virus and drug-induced liver damage and aborted immune responses [55, 56]. In the American College of Cardiology (ACC) report, mortality was higher in COVID-19 patients with pre-existing hypertension, cancer, cardiovascular disease, and diabetes.

With the prevalence of COVID-19, some chemical drugs were gradually put into the COVID-19 treatment. For example, chloroquine and remdesivir have been widely used to prevent SARS virus and COVID-19 [57]. A clinical trial shows that hydroxychloroquine and azithromycin have a dramatic effect on COVID-19 by preventing viral genome replication [58]. Progesterone was first identified in our study, and its many biological functions in the central nervous system have been demonstrated, including regulation of inflammation, mitochondrial function, neurogenesis and regeneration, myelination, and recovery from traumatic injury. Progesterone is an effective inflammation-reducing substance that has been shown to have protective effects on the nervous system. An immune survey uncovers evidence of a regional immune response in the central nervous system of COVID-19 patients and suggests a role for autoimmunity in the neurological sequelae of COVID-19 [59]. Another drug identified was sertraline, which is used to treat depression [60]. Anxiety and depressive symptoms increase during the COVID-19 outbreak [61]. Studies have shown that in vitro stimulation with 8-bromo-cAMP activates the cAMP/PKA pathway [62]. This pathway is critical for neuronal development and growth and plays a key role in CSC regulation [62]. Epoprostenol slows or stabilizes pulmonary vascular disease. In one study, the use of epoprostenol improved survival in patients with severe pulmonary hypertension in WHO functional classes III and IV [63]. In addition, some other drugs, such as losartan, were screened out not only for tumor treatment but also for reducing high blood pressure [63, 64]. Zonisamide is an antiepileptic drug that has been approved for use in many countries, and zonisamide combined with some other drugs can be used to modulate astrocyte Cx43HC function and glial metastasis [65]. These drugs are effective in the treatment of COVID-19, as well as tumors and mental and cardiovascular diseases.

Additionally, there are some limitations in this present study. Our paper is written only based on the public databases using an in silico approach and lacks the validation form for in vivo or in vitro experiments. Therefore, it is necessary to further confirm our findings by in vivo or in vitro experiments in the future.

This study summarizes the relationship between the two disease genes in the context of LC and COVID-19 transcriptome analysis. We conducted differential expression analysis in both data sets, identified overlapping genes, and discovered disease responses between LC-affected and SARS-CoV-2-affected lung cells. These findings showed that LC patients might be at higher risk of contracting COVID-19. Firstly, 32 overlapping genes were identified. Based on overlapping genes, the PPI network was constructed and the 10 most significant hub genes were selected. The hub genes were retrieved through the DSigDB, and multiple drug molecules and drug-target interactions were mined. Our research is a novel approach in identifying the infection of various diseases, especially LC and COVID-19. Study results may help LC patients reduce the risk of being affected by SARS-CoV-2. COVID-19 is a recently popular infectious disease, and its risk factors and associations with other diseases have been particularly poorly studied. With the increase and disclosure of transcriptomic data of COVID-19 patients, this provides an opportunity for researchers to fully study COVID-19. Although there are currently some vaccines that can prevent the infection of the SARS-CoV-2 or alleviate the condition of COVID-19, COVID-19 is still in a serious infection situation worldwide, especially in some countries. Therefore, we performed bioinformatics analysis to detect common pathways and molecular biomarkers between LC and COVID-19, which is of great significance to our understanding of the link between COVID-19 and LC. Ten hub genes were identified to be closely associated with these two types of diseases. All these hub genes play key roles in different functional mutations. The TFs and miRNAs identified in this study are associated with COVID-19. Therefore, these genes we identified might become new therapeutic targets for COVID-19 treatment.

## Data Availability

The data sets used and/or analyzed during the current study are available from the corresponding author on reasonable request.
